# Cathode porosity is a missing key parameter to optimize lithium-sulfur battery energy density

**DOI:** 10.1038/s41467-019-12542-6

**Published:** 2019-10-10

**Authors:** Ning Kang, Yuxiao Lin, Li Yang, Dongping Lu, Jie Xiao, Yue Qi, Mei Cai

**Affiliations:** 1 Optimal CAE, Inc, Plymouth, MI 48170 USA; 2 Department of Chemical Engineering and Materials Science, Michigan State University, East Lansing, MI 48824 USA; 3 Chemical and Materials Systems Laboratory, General Motors Global R&D Center, Warren, MI 48092 USA; 4 Pacific Northwest National Laboratory, Richland, WA 99352 USA

**Keywords:** Energy and society, Scientific community

## Abstract

While high sulfur loading has been pursued as a key parameter to build realistic high-energy lithium-sulfur batteries, less attention has been paid to the cathode porosity, which is much higher in sulfur/carbon composite cathodes than in traditional lithium-ion battery electrodes. For high-energy lithium-sulfur batteries, a dense electrode with low porosity is desired to minimize electrolyte intake, parasitic weight, and cost. Here we report the profound impact on the discharge polarization, reversible capacity, and cell cycling life of lithium-sulfur batteries by decreasing cathode porosities from 70 to 40%. According to the developed mechanism-based analytical model, we demonstrate that sulfur utilization is limited by the solubility of lithium-polysulfides and further conversion from lithium-polysulfides to Li_2_S is limited by the electronically accessible surface area of the carbon matrix. Finally, we predict an optimized cathode porosity to maximize the cell level volumetric energy density without sacrificing the sulfur utilization.

## Abstract (web summary)

For high-energy lithium-sulfur batteries, a dense electrode with low porosity is desired to minimize electrolyte intake, parasitic weight, and cost. Here the authors show the impact of porosity on the performance of lithium-sulfur batteries and reveal the mechanism through analytical modeling.

## Introduction

Increased emphasis on electrification in the automotive industry is driving the need for efficient and compact battery systems. The development of rechargeable batteries with high-energy density and long cycle life continues to be of paramount importance. The conventional insertion compound cathodes used in current lithium-ion (Li-ion) batteries, such as lithium cobalt oxide and lithium iron phosphate, are unable to meet the requirement for the competitive market which has stimulated enormous research for advancing cathodes materials for Li-ion batteries. Of particular interest in this regard is the lithium–sulfur (Li–S) battery which has high theoretical capacity and energy density, as well as the natural abundance and environmental compatibility of sulfur. These very favorable characteristics render Li–S battery technology quite promising for next-generation energy storage devices^1–6^.

Despite its high specific energy density of 2500 kW kg^−1^, which is considerably higher than traditional Li-ion batteries, as well as the low cost, the practical application of Li–S battery is still challenging. The major issues facing its broader applications are: the intrinsic insulating characteristics of sulfur, the shuttle phenomenon that results from the high solubility of lithium polysulfide (Li-PS), the volume expansion of sulfur during lithiation, and the highly reactive Li-metal surface induced side reaction and mossy/dendrite growth^7–9^. To address these issues, host materials have been incorporated with elemental sulfur to increase the electrical conductivity and sulfur utilization. Carbonaceous materials, such as mesoporous carbon and microporous carbon, have been demonstrated with improved sulfur utilization and cycle stability due to large pore volume and the reduction of Li-PS diffusion^10,11^. Furthermore, carbon hosts with tailored nanostructures have also been explored^12–14^, to increase electrical conductivity with additional electron pathways and interconnected ion diffusion channels. Alternatively, transition metal oxides, such as TiO_2_, MnO_2_ etc., have also been investigated as host materials because of their strong affinity to Li-PS^15^. In addition to the host materials, polymer binders, such as polyethylene oxide (PEO) and carboxymethyl cellulose styrene (CMC), etc.^16–22^, as well as porous current collectors (Al, carbon)^23–25^, have also been extensively explored to improve the performance of Li–S battery. The degradation of lithium metal anode also hinders the commercialization of Li–S batteries. The lithium dendrite accompanied by the formation of solid electrolyte interphase (SEI) not only leads to capacity loss but also raises safety concerns. It has been proposed that the lithium surface can be protected by an artificial SEI layer via in vitro coating deposition or by controlling the formation of the SEI through in vivo electrolyte/additive design^11,26–30^. Due to continuous lithium stripping and plating in Li–S batteries, electrolyte with lower reduction potential, has been demonstrated to facilitate less formation of SEI^31–34^. LiNO_3_ can effectively passivate lithium and prevent the electrochemical reduction of Li-PS on the surface of lithium for Li–S batteries^35^. However, a carefully controlled discharge cutoff voltage is needed to prevent it from damaging other components, such as cathode binders, in the battery^36,37^.

The infiltration of sulfur into a porous carbon matrix as a sulfur/carbon (S/C) composite for a sulfur electrode is considered as an effective approach that can be easily scaled up to suppress the diffusion of Li-PS and improve the transport of electrolyte^38,39^. The pore size and structure play an important role in the electrochemical performance of Li–S batteries due to the Li-PS dissolution associated redox shuttle reactions. Because of the depletion of electrolyte, most cells were assembled and tested with excess electrolyte, namely electrolyte/sulfur (E/S) ratio >10 μL mg^−1^. The amount of electrolyte is critical for the volumetric energy density of batteries, since it accounts for a major part of the total cell weight^40^. At present, the state of art designs for Li–S pouch cell requires an E/S ratio of 3 μL mg^−1^ to achieve a gravimetric energy density of 300 Wh kg^−1^ which is comparable to commercially available Li-ion battery^41^. Besides, electrolyte quantity is strongly associated with the porosity of the electrode. The cell level gravimetric energy density will be around 400 Wh kg^−1^ with 70% porosity when the areal capacity of sulfur is 5 mAh cm^−2^. If the porosity of the electrode is reduced by 10%, the gravimetric energy density may reach 500 Wh kg^−1^ due to the reduction of electrolyte quantity. This is the target of the Department of Energy Battery 500 program for the advanced battery of electric vehicles^42^. Inspired by the above insights, manipulating the porosity to obtain Li–S battery with stable electrochemical performance and higher gravimetric energy density (≥500 Wh kg^−1^) is of immense importance.

In this study, a combined experimental/theoretical approach was developed to quantify the effects of sulfur electrode porosity on Li–S batteries. First, the electrochemical performance of the sulfur electrodes with different porosities is examined. Specifically, the relationship between the energy density and the porosity is determined and utilized to predict the porosity to achieve a gravimetric energy density of 500 Wh kg^−1^. The porosity design leads to an optimized design of Li–S battery and make it more attractive for the niche market.

## Results

### Morphology of sulfur electrode with different porosity

**Fig. 1:**
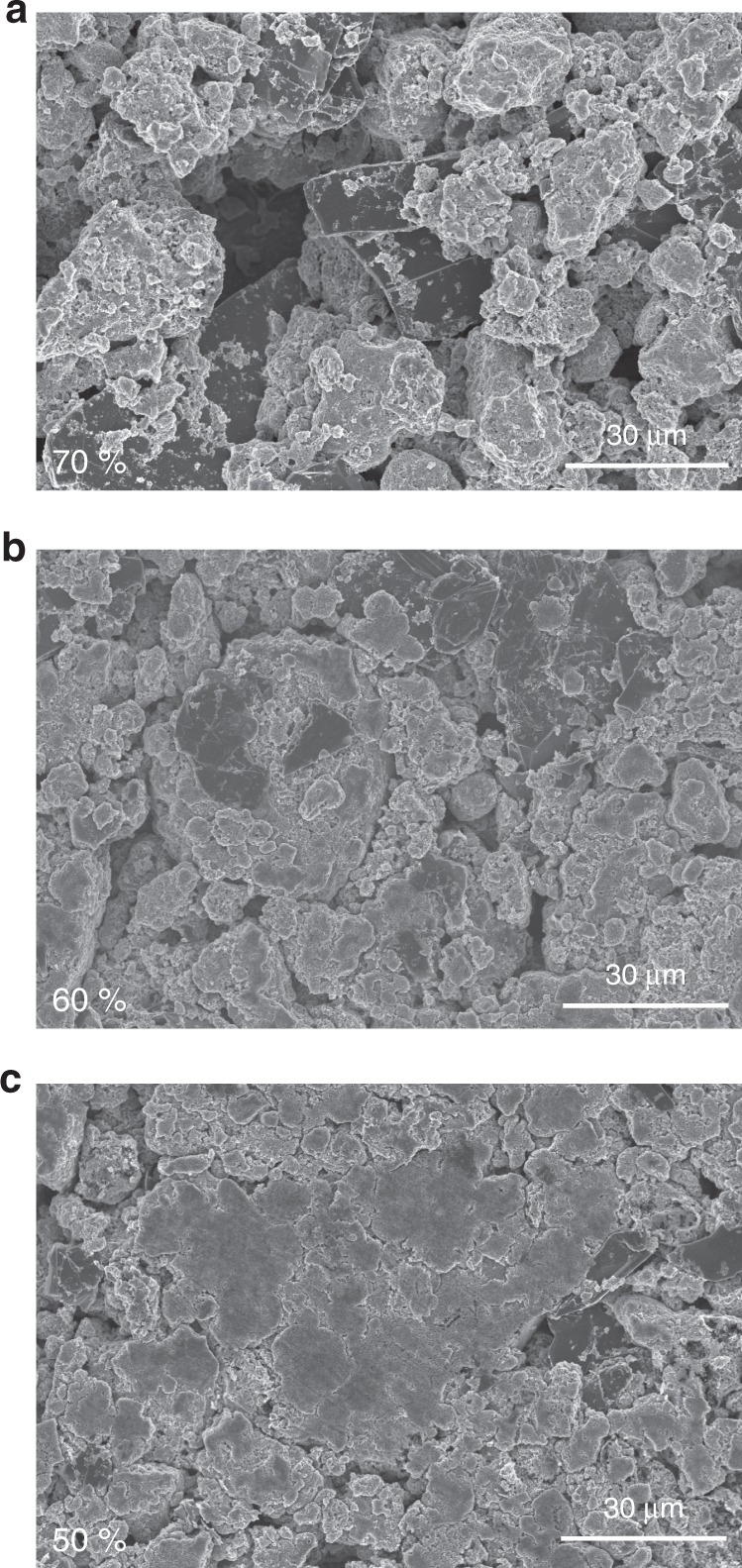
Scanning electron microscope (SEM) images. SEM images of the sulfur electrode with a porosity of 70, 60, and 50%

The morphology of S/C composite electrodes with different porosities is observed with scanning electron microscopy (SEM), as it shows in Fig. 1. At a porosity of 70%, large void space is witnessed with particles overlapped and distributed nonuniformly across the surface. At a porosity of 60% or 50%, the distance between particles was greatly reduced and the empty space was also less visible due to the calendering process. Meanwhile, particles packed more condensed across the surface of the electrode, and the aggregation is also obvious. The morphology of the sulfur electrode with the porosity of 40 and 50% after cycling was observed with cross-section SEM images (Supplementary Fig. 1 in the Supporting Information, SI). The thickness of both electrodes increases due to the volume expansion during cycling. The carbon surface is also covered by deposited materials, likely to be the insulating Li_2_S.

### Electrochemical performance of cells with different porosity

For the sulfur cathode, a high fraction of pore volume is essential to provide space for both redox reactions and volume expansion of sulfur (~80%)^40,43^. At the same time, some excess electrolyte is required to fully fill the pores to ensure interconnected Li-ion transfer pathways between particles. The initial porosity of the as-coated electrode is 70%. Through a calendering process, electrodes with various porosities were prepared for electrochemical performance evaluation.

**Fig. 2:**
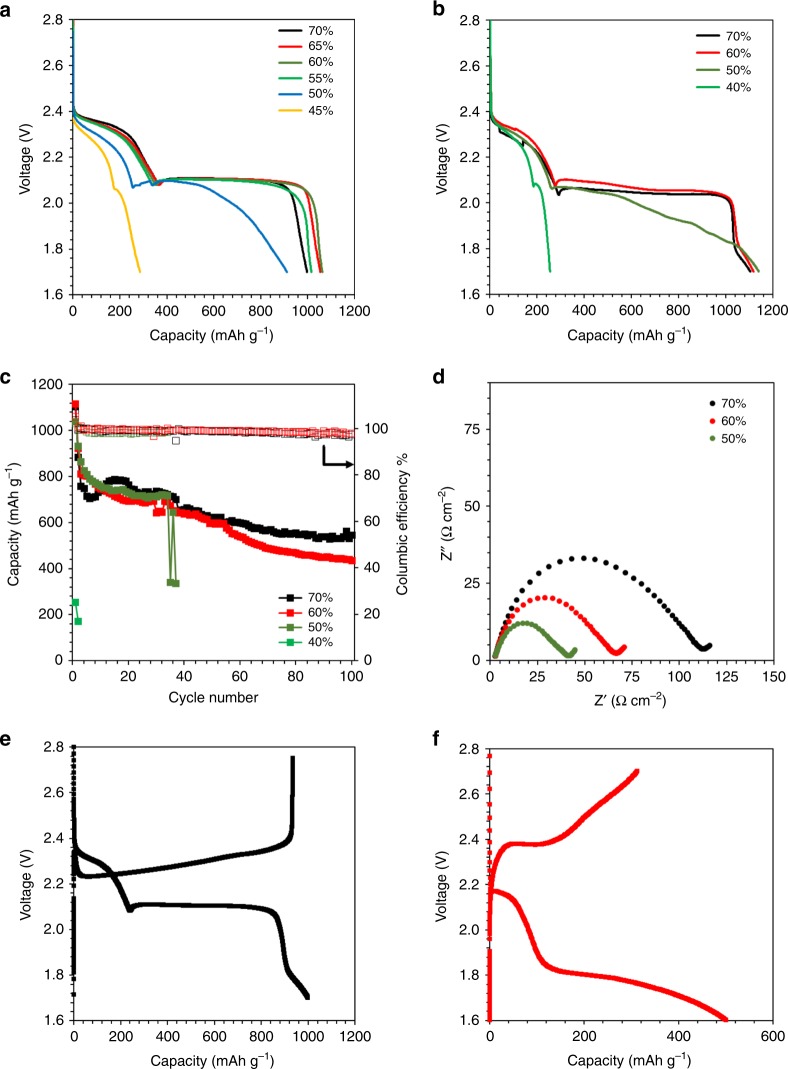
Electrochemical performance of cells with different sulfur mass loading. **a**, **b** Charge–discharge profiles of lithium–sulfur (Li–S) cells with sulfur loading of 2.5 mg cm^−2^ and 5 mg cm^−2^ under different porosity. **c** Cycle performance of cells with the sulfur loading of 5 mg cm^−2^ under porosity of 70, 60, 50, and 40%. **d** Electrochemical impedance spectrum of sulfur electrode with porosity of 70, 60, and 50% (sulfur loading of 2.5 mg cm^−2^). **e**, **f** Li–S battery with electrolyte composed of dimethoxyethane (DME) and dioxolane (DOL) as solvent with 0.4 M lithium bis(trifluoromethanesulfonyl)imide (LiTFSI)−0.6 M LiNO_3_ and 3 M LiTFSI–0.2 M LiNO_3_ (sulfur loading of 2.5 mg cm^−2^)

Figure 2a shows the charge–discharge profiles of Li–S cells with a sulfur loading of 2.5 mg cm^−2^ at the porosities of 70, 65, 60, 55, 50, and 45%. A typical two-plateau discharge behavior of Li–S battery was observed when the porosity was higher than 55%. The first plateau at ~2.4 V is generally ascribed to the conversion reactions of elemental S to soluble Li-PS (including Li_2_S_8_, Li_2_S_6_, and Li_2_S_4_), and the second 2.1 V plateau indicates further conversion of soluble Li-PS to insoluble Li_2_S_2_ and Li_2_S. The turning point remained constant at a capacity of about 360 mAh g^−1^, and the total initial discharge capacity was above 1000 mAh g^−1^ for cells with porosity higher than 50%, implying that sulfur utilization rate was not severely affected when the porosity was within a certain range. As the porosity was further reduced to 50%, the plateau turning point moved backward to 260  mAh g^−1^, and significant depression of the second plateau was observed. The total initial capacity decreased to 910 mAh g^−1^. This phenomenon was even more severe for the cell with a lower porosity of 45%. The turning point was further reduced to a capacity of 200 mAh g^−1^, and the total capacity decreased sharply to only 299 mAh g^−1^. A similar trend can also be observed for the electrodes with sulfur loading of 5 mg cm^−2^, as shown in Fig. 2b. Electrodes with porosities of 70 and 60% showed identical capacities close to 1100 mAh g^−1^ with the second discharge plateau above 2.0 V. Though the 50% porosity electrode delivered relatively higher initial capacity compared with the lower loading electrode at the same porosity, a significantly depressed second discharge plateau was still observed. When the porosity was further reduced to 40%, the initial discharge capacity is only 255 mAh g^−1^, and the 2^nd^ discharge plateau was compressed severely. These results indicated that electrodes with higher porosity can provide more reaction sites and thus have higher sulfur utilization rate.

The cycle performance of the cells with sulfur loading of 5 mg cm^−2^ under different porosities is presented in Fig. 2c. The electrodes with porosities of 70, 60, and 50% show very close initial capacities of 1104, 1116, and 1040 mAh g^−1^, respectively. The capacity of the 70% porosity electrode decreased in the first 12 cycles, and then recovered and maintained relatively stable. The cell with a porosity of 60% showed a slightly higher capacity for the first 10 cycles compared with the 70% porosity electrode, but the capacity decayed more significantly in the following cycles. The cell with a porosity of 50% presented more stable cycle performance compared with cells with higher porosities, but the capacity dropped suddenly after 34 cycles which may be caused by short-circuiting. As for the cell with a porosity of 40%, it failed quickly after two cycles with an initial capacity of 255 mAh g^−1^. Though small pores of the carbon matrix are beneficial for the utilization of active materials^40^, the electrode with a porosity of 70% still presented better performance compared with the ones with lower porosities. At low sulfur loading (Supplementary Fig. 2), the cell with a porosity of 70% showed a stable cycle performance, although the initial discharge capacity was lower than the cells with lower porosities (65, 60, and 55%). While the initial capacity of the cell with a porosity of 60% was the highest among all the cells, it kept decreasing and almost equaled to the one with a porosity of 65% after 10 cycles. Both cells with the porosity of 70 and 55% experienced fast capacity decay after 20 cycles compared with cells with porosity of 60 and 65%. The capacity of the cell with a porosity of 65% gradually increased till 80 cycles, then suddenly dropped to an even lower capacity than the cell with the porosity of 60 and 55%. The cell with a porosity of 70% showed most stable cycle performance for 100 cycles compared with others, followed by 60 and 55%. The Coulombic efficiency of all the cells maintained around 98%, which could be attributed to inhibition of shuttle effect by the utilization of LiNO_3_^35,44^. The decomposition of LiNO_3_ is also indicated by the small extra capacity gain when the voltage is below 1.8 V for porosity larger than 50% in Fig. 2b, consistent with the observations in the literature^35^.

Figure 2d shows the electrochemical impedance spectroscopy for cells with electrode porosities of 70, 60, and 50% after 5 cycles at 50% state of charge. As porosity decreased by 10%, the cell charge transfer resistance decreased almost by a half while the resistance of electrolyte remained the same, indicating that calendering process could enhance the conductivity through improving the contact between particles^45^. However, the calendering process also had a negative effect on the electrochemical performance of the cells resulting from the significant reduction of void space and pore volume, as discussed in the above context.

The poor electrochemical performance of sulfur electrode with reduced porosity demonstrates that the proportion of pore volume is crucial for both sulfur utilization rate and cycling stability. Unlike conventional intercalation Li-ion battery, the reaction mechanism of the Li–S battery involves Li-PS dissolution and deposition processes, which is highly dependent on the electrolyte properties. As shown in Fig. 2e, with low concentration electrolyte, the cell presented a long 2^nd^ discharge plateau at 2.1 V, which, however, was compressed remarkably if the concentration was increased to 3 mol L^−1^ (M, see Fig. 2f). It was evident that the electrochemical performance of Li–S battery was strongly affected by the availability of the free solvents in the electrolyte which probably related to the dissolution of Li-PS. If the concentration of lithium Li-PS in the cell is beyond the limit of its solubility (~8 M), the performance of the battery will be negatively affected^46^.

### Analytical model

According to the experimental observation, the overall performance of the Li–S battery was highly impacted by the porosity of the S/C composite cathode. It has been reported that the surface area of the carbon of the S/C cathodes had a significant impact on the second discharge plateau^47^. Our experimental data showed the decrease of the porosity not only caused a depressed second plateau, but also a shortened first plateau as shown in Fig. 2a, b. To understand the fundamental mechanisms, a unified analytical model was constructed to quantify the impact of cathode porosity, *p*, on the cell-level energy density. In the experiments, we have chosen flooded coin cells with baseline electrolyte to decouple the influence of cathode porosity from other experimental factors, such as electrolyte consumption due to SEI formation on Li-metal surface. Due to the flooded coin cell design, not all the electrolyte was utilized. Our analytical model will rationalize the amount of electrolyte participate in cell operation and Li-PS dissolution. This will serve as a base to estimate the limit of E/S ratio in practical pouch design with no excess electrolyte.

### Limited capacities in the first discharge plateau

After cell assembly, the electrode pores shown in Fig. 1 will be filled with electrolyte. Thus, the amount of electrolyte required for full electrode wetting will scale with the electrode porosity, *p*. Considering porous polymer separator and non-porous Li anode used for the cell fabrication, the total pore volume of the cell, *V*_pore_, included the pores in the separator and the cathode, as

1 $$V_{{\mathrm{pore}}} = V_{{\mathrm{pore}}}({\mathrm{sep}}) + V_{{\mathrm{pore}}}\left( {{\mathrm{cat}}} \right)$$

*V*_pore_(sep) was calculated to be 2.5 mm^3^ based on the total volume (6.3 mm^3^) and the porosity (40%) of the Celgard 2500 separator used in the experiment. *V*_pore_(cat) included the micropores inside the carbon and the void space between carbon particles. It scaled with the porosity, *p* as

2 $$V_{{\mathrm{pore}}}\left( {{\mathrm{cat}}} \right) = p \ast V\left( {{\mathrm{cat}}} \right) = p(V_{{\mathrm{dense}}}\left( {{\mathrm{cat}}} \right) + V_{{\mathrm{pore}}}\left( {{\mathrm{cat}}} \right))$$

Based on the experimentally measured compact volume of the cathode, the volume of dense S/C composite cathode without any porosity *V*_dense_(cat) was about 5.3 mm^3^ (Supplementary Table 1). First, we can assume all the electrolyte can be utilized. The diffusion coefficients of the smallest Li-PS, Li_2_S_4_, in a typical solvent dimethoxyethane (DME), were calculated as a function of Li_2_S_4_ concentrations by classical molecular dynamics (MD) simulation. Although the Li_2_S_4_ diffusion coefficient decreased with increasing concentration, the diffusion distance of Li_2_S_4_ during the discharging process at a rate of 0.1 C was estimated to be 1–3 mm which is far beyond the pore and particle size of the S/C composite and electrode thickness. This supports the occurrence of Li-PS shuttling, which was widely observed in published experiments^10,11^. It also suggested that the diffusion of Li-PS was not the limiting factor for sulfur utilization.

Based on the sulfur loading of 5.0 mg cm^−2^, the total mass of S in the cathode, *m*_s_(total) was 6.5 mg for a cathode with an area of 1.3 cm^2^, corresponding to a volume of 3.25 mm^3^. (Supplementary Table 1). If all the 6.5 mg S had converted to Li_2_S_4_ after completion of the first discharge plateau, a theoretical capacity of *Q*_th_ = 420 mAh g^−1^ would have been obtained. As all the experimentally measured discharge capacity after the first plateau is less than *Q*_th_, we introduced S utilization percentage *P*_s_(uti), as the ratio of the mass of utilized S (those converted to Li_2_S_4_), *m*_s_(uti), and the total mass of S as

3 $$P_s({\mathrm{uti}}) = \frac{{m_{\mathrm{s}}\left( {{\mathrm{uti}}} \right)}}{{m_{\mathrm{s}}\left( {{\mathrm{total}}} \right)}} = \frac{{Q_{{\mathrm{pr}}}}}{{Q_{{\mathrm{th}}}}}$$

**Fig. 3:**
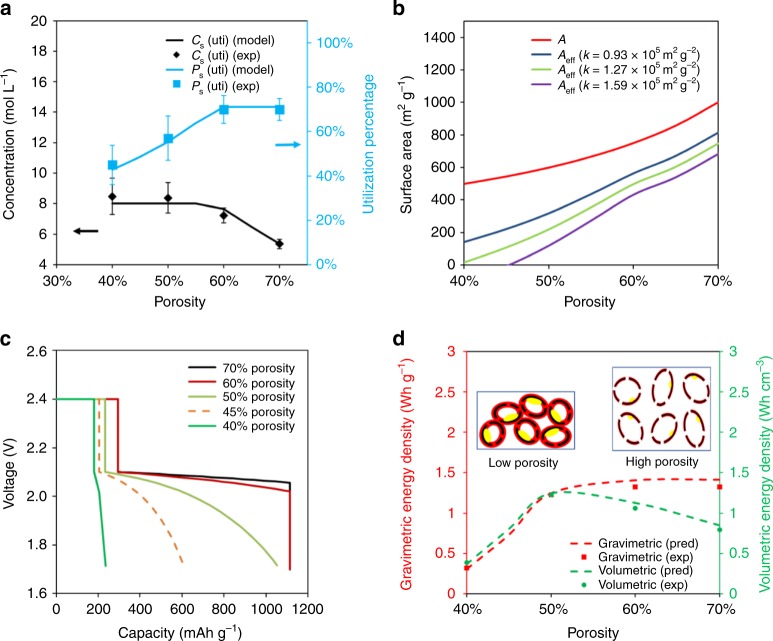
Analytical modeling results. Predicted lithium-polysulfide (Li-PS) concentration and sulfur utilization as a function of porosity (**a**). Predicted total surface area and effective surface area as a function of porosity (**b**). Predicted discharging curves at different porosity (**c**). The predicted gravimetric energy density based on the total mass of the cathode including S, carbon matrix, carbon black and carboxymethyl cellulose and styrene-butadiene rubber (CMC-SBR), and volumetric energy density based on the comprehensive volume of the cathode (**d**). The schematics showing the difference between low porosity and high porosity were also inserted. The unutilized sulfur, carbon matrix, and deposited Li_2_S_2_/Li_2_S layer were represented as yellow, black, and red, respectively

*Q*_pr_ was the practical capacity in the first plateau, which could be obtained from Fig. 2b. As shown in Fig. 3a, the experimental *P*_s_(uti) was maintained at ~70% for porosity higher than 60%. Thus, at most 70% of S could be converted to Li_2_S_4_. The Li_2_S_4_ solubility in the electrolyte solvent in terms of S is *C*_max_ = 8 M^19^. Assuming these Li_2_S_4_ was only dissolved into the electrolyte in the pore, its concentration would still exceed the saturation limit even at a high porosity of 70%. That means the accessible electrolyte volume was larger than *V*_pore_. Further decrease in porosity below 60% led to a reduction of *P*_s_(uti), since the amount of accessible electrolyte no longer fully dissolve the produced Li_2_S_4_, thus limits the utilization of active S. Therefore, we consider the first plateau will be terminated once the Li_2_S_4_ concentration reaches the saturation limit, thus the S utilization will be limited by the maximum amount of soluble Li_2_S_4_,

4 $$P_{\mathrm{s}}({\mathrm{uti}}) = \frac{{gV_{{\mathrm{pore}}}M_{\mathrm{s}} \cdot C_{{\mathrm{max}}}}}{{m_{\mathrm{s}}\left( {{\mathrm{total}}} \right)}}$$

where *M*_s_ = 32 g mol^−1^ is the molar mass of S and the accessible electrolyte volume was considered as *gV*_pore_. A new parameter *g* was introduced to account for the accessible electrolyte outside of the pores but contributed to Li-PS dissolution. By fitting to the practical capacity of the first plateau at different porosities as shown in Fig. 2b, the value of *g* was determined to be 1.8, suggesting the volume of utilized electrolyte is about twice (1.8 times) of that inside of the pore.

Based on the amount of utilized sulfur, the dissolved Li_2_S_4_ concentration was also calculated in terms of the mole of S.

5 $$c_{\mathrm{s}}\left( {{\mathrm{uti}}} \right) = \frac{{m_{\mathrm{s}}\left( {{\mathrm{uti}}} \right)}}{{gV_{{\mathrm{pore}}}M_{\mathrm{s}}}}$$

In Fig. 3a, *c*_s_(uti) increased with reduced porosity and converged at the saturated concentration of 8 M^19^ when the porosity was below 60%. This confirmed the saturation of Li-PS in the electrolyte solvent was the limiting factor for the capacity in the first plateau.

### Depression of the second discharge plateau

The electrochemical reaction corresponding to the second plateau will proceed on the carbon surface, where the dissolved Li_2_S_4_ takes electrons from the surface and Li-ion from the electrolyte and deposits as insulating Li_2_S_2_ and Li_2_S on the surface.

The depression of the second plateau is related to the loss of the pore surface area, *A*, which also scales with cathode porosity, *p*, according to different pore structure models^48^. In general, the total surface area, $A \propto V({\mathrm{cat}})^\alpha$, where *α* is an empirical constant depending on the pore structure, usually in the range from −1 to 1. Given the fact that the porosity was changed by the calendering process along the thickness direction, the *α* value of 1 was chosen. Further relating *V*(cat) with the porosity *p*, the total surface area *A* decreased with *p*

6 $$A(p) = \frac{{A_0}}{{1 - p}}$$

As we know the measured Brunauer–Emmett–Teller (BET) surface area, *A*, was about ~1000–1100 m^2^ g^−1^ at *p* = 70%, the rest of A can be obtained as a function of *p*.

If all the sulfur can be converted to Li_2_S_4_ dissolved in the electrolyte, the carbon surface originally covered by the insulating sulfur will become available again to facilitate the charge transfer reaction. However, if there is unutilized S left after the reaction that associated with the first plateau, part of the surface that covered by S is still insulating. Thus, an effective surface area, *A*_eff_, was defined to account for the unutilized sulfur, $m_{\mathrm{s}}\left( {{\mathrm{total}}} \right)(1 - P_{\mathrm{s}}\left( {{\mathrm{uti}}} \right))$, with a parameter of *k*,

7 $$A_{{\mathrm{eff}}} = A-km_{\mathrm{s}}\left( {{\mathrm{total}}} \right)(1 - P_{\mathrm{s}}\left( {{\mathrm{uti}}} \right))$$

Knowing the sulfur utilization percentage, assuming different *k*, *A,* and *A*_eff_ were plotted as a function of *p* in Fig. 3b. Both *A*_0_ and *A*_eff_ decreased with the reduction of porosity, but the decrease in *A*_eff_ was much faster compared with *A*. This was due to the increase of unutilized sulfur covering carbon surface area. The exact value of *k* will be determined later.

Assuming the deposited insulating Li_2_S_2_/Li_2_S products are uniformly distributed on the carbon surface, the layer thickness *d* is proportional to the capacity in the second plateau $Q - Q_{{\mathrm{th}}}P_{\mathrm{s}}\left( {{\mathrm{uti}}} \right),$ and inversely proportional to the effective surface area *A*_eff_. The *Q* here was the total capacity. Thus, using a constant *b*, a relationship between thickness *d* and total capacity Q can be derived as

8 $$d = b\frac{{(Q - Q_{{\mathrm{th}}}P_{\mathrm{s}}\left( {{\mathrm{uti}}} \right))}}{{A_{{\mathrm{eff}}}m_{\mathrm{C}}}}$$

*m*_c_ = 1.85 mg was the mass of the carbon matrix used in the experiment. The insulating Li_2_S_2_/Li_2_S layer will induce the resistance and the electrons must tunnel through its thickness to continue the electrochemical reactions. The tunneling resistance *R* would increase exponentially with the thickness *d* of the insulating layer^49^, as the following

9 $$R = C({\mathrm{e}}^{Bd} - 1)$$

where *C* and *B* were two fitting parameters, which will be discussed later. According to Eq. (9), *R* was 0 when the thickness *d* was negligible. The insulating layer induced *IR* drop, with *I* representing the discharging current, in the discharge curve for the second plateau. The uniform deposition of insulating Li_2_S_2_/Li_2_S on the carbon surface was supported by the S/C electrode produced by melt-diffusion method (similar to ours) by Pan et al.^50^. Interestingly, they also introduced a non-uniform Li_2_S deposition, a so-called ‘flower-like’ Li_2_S agglomeration, which kept the carbon fiber and sulfur cathode electrochemically active. It will be interesting if it can be achieved in a macroporous carbon matrix.

Taking the equilibrium open circuit voltage of 2.4 and 2.1 V for the first and second plateau, the discharge curve will show two stages for voltage *V* and capacity *Q*:

10 $$V = \left\{ {\begin{array}{*{20}{c}} {2.4\ \ \ \ \ \ \ \ \ \ \ \ \ \ \ \ \ \ \ \ \ \ \ \ (0 \ < \ Q \ < \ Q_{{\mathrm{th}}}P_{\mathrm{s}}\left( {{\mathrm{uti}}} \right))} \\ {2.1 - C^\prime \left( {{\mathrm{e}}^{\frac{{B^\prime (Q - Q_{{\mathrm{th}}}P_{\mathrm{s}}\left( {{\mathrm{uti}}} \right))}}{{A_{{\mathrm{eff}}}m_{\mathrm{C}}}}} - 1} \right)\left( {Q \ > \ Q_{{\mathrm{th}}}P_{\mathrm{s}}\left( {{\mathrm{uti}}} \right)} \right)} \end{array}} \right.$$

*B*′ *=* *Bb* and *C*′ *=* *CI* were two combined parameters. By fitting the discharge curves with 40 and 50% porosity in Fig. 2b, the fitted value of *B*′*, C*′, and *k* were determined as 1.07 × 10^–3^ m^2^ g mAh^−1^, 0.050 V and 1.27 × 10^5^ m^2^ g^−2^, respectively. Although we did not explicitly count the cathode swelling shown in Supplementary Fig. 1, the excess electrolyte volume in Eq. (1) and the fitting parameters in Eq. (2) should have implicitly included the swelling effect. The predicted discharging curves at other porosities were shown in Fig. 3c. The model successfully predicted the discharging curves by negligible changes when the porosity was higher than 60%. Further decrease in porosity shortened the first plateau and depressed the second plateau. Thus, the capacity dropped rapidly when the porosity decreases to the range of 40–50%. This was also consistent with experimental observation.

In addition, this analytical model can also explain the open circuit voltage change from Fig. 2e to Fig. 2f when the lithium bis(trifluoromethanesulfonyl)imide (LiTFSI) concentration in the electrolyte increased from 1 to 3 M. Similar to the “water in salt” system^51^, with an increase in the LiTFSI concentration, the amount of free solvent that can be used to solvate Li-PS will drop. This led to a lower Li-PS saturation concentration in the electrolyte and a reduction in *P*_s_(uti), which shortened the first plateau and depressed the second plateau as well. This is the reason why even at a high porosity of 70%, the electrochemical performance was greatly affected by 3 M LiTFSI electrolyte in Fig. 2f.

### The volumetric and gravimetric energy density

Based on the above discussion, the impact of porosity on the electrochemical performance can be summarized in the schematics in Fig. 3d. The unutilized S, carbon matrix and deposited Li_2_S_2_/Li_2_S layer were represented as yellow, black and red, respectively. When the porosity was high (>55%), *P*_s_(uti) maintained at 70%, contributing to a long first plateau. Besides, *A*_eff_ was at least 500 m^2^ g^−1^, which was high enough to keep the deposited Li_2_S_2_/Li_2_S layer thin until all generated Li-PS was converted to Li_2_S_2_ and Li_2_S, as shown in the right schematics in Fig. 3d. The resistance caused by this thin layer formed on the carbon surface was less than 0.1 V and the second plateau would remain flat. With a medium porosity (~50%), *P*_s_(uti) dropped to 55%, leading to a shortened first plateau. Furthermore, due to the decrease in porosity and the increase in unutilized sulfur, *A*_eff_ was only ~200 m^2^ g^−1^. The deposited Li_2_S_2_/Li_2_S layer grew thicker as the second discharging reaction continued, causing the increase in the resistance. As shown by the analytical model, the IR drop was ~0.2 V at a total capacity of 800 mAh g^−1^ and ~0.4 V at a total capacity of 1100 mAh g^−1^, demonstrating an obvious depression in the second plateau. At low porosity (<45%), *P*_s_(uti) of ~42% led to an even shorter first plateau. *A*_eff_ also dropped dramatically to ~15 m^2^ g^−1^. As a result, the thickness of the Li_2_S_2_/Li_2_S layer and the corresponding resistance grew very quickly, as shown in the left inserted schematics in Fig. 3d. The total capacity was <250 mAh g^−1^ when the *IR* dropped 0.4 V and the voltage limit of 1.7 V was reached. This is the reason for the severe depression of the second plateau accompanied by low porosity.

In Fig. 3d, the predicted gravimetric energy density was calculated as the total energy (calculated from the discharging curve) divided by the total mass of cathode. The volumetric energy density was calculated as the total energy divided by the comprehensive volume of the cathode, *V*(cat) in Eq. (1). The gravimetric energy density first increases monotonically with increasing porosity and reached a constant when the porosity is above 55%. The volumetric energy density, however, showed a peak value around 52% porosity.

### Critical parameters for cell-level design

**Fig. 4:**
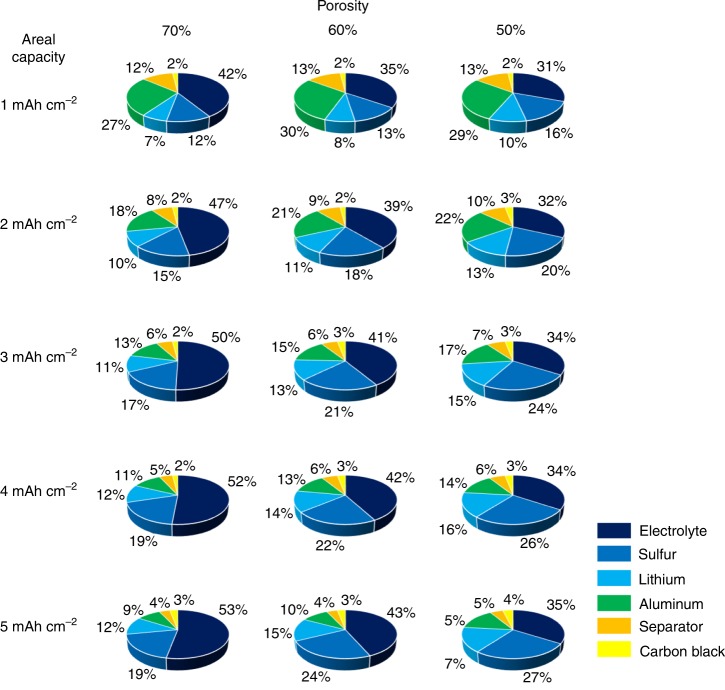
Parameters for cell-level design. Electrolyte/sulfur (E/S) ratio in pouch cell with different porosity under various sulfur areal capacity

Although excess electrolyte could increase the utilization of sulfur and enhance the cycle performance, it is not an effective approach to achieve high gravimetric energy density. Recently, some promising performance has been demonstrated in Li–S coin cells with E/S ratio of <5 μL mg^−1^ under specific conditions^52–54^. As discussed above, for practical application the recommended E/S ratio is 3 μL mg^−1^ that is comparable to the commercially available Li-ion battery^52^. Supplementary Table 2 illustrates the E/S ratio values at a different areal capacity and porosity based on coin cells considering the pore volume in the sulfur electrode and separator. The E/S ratio typically decreases with areal capacity and porosity. When the areal capacity of sulfur is 5 mAh cm^−2^, the E/S ratio is close to 4 μL mg^−1^ even for an electrode with porosity of 70%, and it becomes even smaller as porosity decreases. In the pouch cell format, the E/S ratio could be further reduced due to the elimination of dead space and uneven distribution of pressure^10^. As shown in Fig. 4, the proportion of electrolyte increases from 42 to 53% when the areal capacity of sulfur changes from 1 to 5 mAh cm^−2^ at a fixed porosity of 70%. Such extremely high electrode porosity reduces energy density and adds cost due to the large amount of electrolyte. If the areal capacity of sulfur electrode maintains the same, the utilization of electrolyte quantity decreases with the reduction of porosity. For electrodes with areal capacity of  5 mAh cm^−2^, the proportion of electrolyte is reduced by 18% when the porosity is decreased to 50%. This phenomenon indicates that porosity plays a significant role in overall cell design because it determines the electrolyte quantity in the cell. However, the cell electrochemical performance may deteriorate if the electrode porosity is decreased below a level of 50% as demonstrated in Fig. 3c. In Fig. 3d, a maximum volumetric energy density was predicted by the analytical model, suggesting that a porosity between 50 and 60% is optimal for a balanced sulfur utilization and cell-level energy density for the given sulfur loading.

Thus, the analytical model enables cell-level Li–S battery design. To utilize this model for other cell designs, such as different separator or electrolyte, the measured constants listed in Supplementary Table 3 may be adjusted accordingly and the fitting parameters {*g*, *B*′, *C*′, and *k*} need to be refitted, while the key equations remain the same. The model can then be used to optimize cathode porosity to maximize cell energy density. It should be noted that cathode porosity is a key parameter in high-energy Li–S cell design but not the only one. To describe the microstructure of the electrode, mesoscale modeling with more specific and accurate parameters, as reviewed by Ryan and Mukherjee^55^, is needed in future work. The current analytical model also did not consider modeling cell cycle life, which requires a degradation mechanism-based model, where electrolyte side reaction and Li anode aging should also be considered.

## Discussion

Effects of electrode porosity on sulfur utilization rate, discharge plateau and cell energy have been studied and discussed. When sulfur loading was 5 mg cm^−2^, the electrode with porosity above 60% showed a stable second discharge plateau and an initial discharge capacity close to 1100 mAh g^−1^. The second discharge plateau was depressed if the electrode porosity is reduced to 50% or less. For electrodes with porosity < 40%, the corresponding second discharge plateau almost disappeared and overall initial discharge capacity is limited to 255 mAh g^−1^. Though cell with a porosity of 60% showed a slightly higher capacity than the one with a porosity of 70%, the latter presented more stable cycle performance as well as higher capacity retention after 100 cycles. At sulfur loading of 2.5 mg cm^−2^, a similar trend was observed for cells with porosity lower than 55% when a severe depressed 2^nd^ discharge plateau and low initial discharge capacity were noticed. An analytical model was built to investigate the effects of electrode porosity and a Li-PS saturation limiting mechanism was suggested based on the theoretical calculation and experimental results. As the electrode porosity decreases, the concentration of the utilized sulfur increases and converges at the saturation limit of 8 M, which is the limiting factor for the first plateau. The loss of the effective surface area due to the deposition of insulating Li_2_S_2_/Li_2_S products is the limiting factor for the 2^nd^ plateau. The galvanic charge–discharge curves from the analytical model corresponded well with the experimental results for the electrodes with different porosities. Our analytical model predicted that the cell reaches a maximum energy density at an electrode porosity of 52% without sacrificing sulfur utilization rate, which is consistent with the experimental observation. Therefore, electrodes with the porosity of 50–60% are suggested for a practical high-energy Li–S cell design.

## Methods

### Electrode preparation

The sulfur cathode material used in the present work is integrated Ketjen Black/sulfur (IKB/S) composite. The detailed description for the synthesis of IKB was reported in our previous work^56^. The IKB/S composite was prepared by melt-diffusion approach. Typically, 80 wt% of sulfur powder (Sigma-Aldrich) was mixed with the synthesized IKB (20 wt%) by hand mill, then the mixture was transferred to Teflon-lined stainless steel autoclave and heat treated at 155 °C for 12 h to improve the sulfur distribution inside the carbon framework. The IKB has a BET surface area of ~1148 m^2^ g^−1^, and a total pore volume of 3.08 cm^3^ g^−1^ before sulfur loading. The measured BET surface and pore volume after sulfur loading (80 wt%) are 12 m^2^ g^−1^ and 0.15 cm^3^ g^−1^, respectively (Supplementary Fig. 3).

In this study, electrodes with sulfur loading of 2.5 and 5 mg cm^−2^ were prepared using slurring coating method. The sulfur/IKB was first prepared from pre-infiltration of elemental sulfur into porous carbon in a vacuum sealed tube at 200 °C for 2.5 h with a ratio of 70:20 (or 78 wt% of sulfur). Then, the sulfur electrode was prepared with S/IKB-carbon matrix composite, carbon black, carboxymethyl cellulose, and styrene-butadiene rubber (CMC-SBR) with a weight ratio of 76:18:6. The weight percentage, mass, compact density, and compact volume of all the materials used in the cathode were listed in Supplementary Table 1.

Deionized water was added into the above mixture to form a uniform slurry with a solid content of 30–40%. CNT (carbon nanotube in H_2_O, 0.2% Sigma-Aldrich) was added to further increase the conductivity which usually accounts for 0.02–0.2% of the resulting slurry. The slurry was finally coated on aluminum foil. The coated sulfur electrode was first dried in the air, then punched into circular pieces with an area of 1.327 cm^2^, followed by vacuum dry at 60 °C overnight in the ambient chamber of the glovebox. The calendering process was performed on a Hohsen Table Top Heat Roll Press instrument at 50 °C. By adjusting the gap between the rollers, electrodes with different porosity were prepared. The porosity, *p*, is defined as

11 $$p = \frac{{V\left( {{\mathrm{cat}}} \right) - V_{{\mathrm{dense}}}\left( {{\mathrm{cat}}} \right)}}{{V\left( {{\mathrm{cat}}} \right)}}$$

where *V*_dense_(cat) is the dense volume of the cathode (5.31 mm^3^) listed in Supplementary Table 1, and *V*(cat) is the comprehensive volume of the electrode, which is determined by the area of the cathode multiplied by the thickness of the cathode after calendaring. Therefore, the thickness of the cathode of 140, 100, 80, and 60 μm corresponds to the porosity of 70%, 60%, 50%, and 40%, respectively, for a sulfur loading of 5 mg cm^−2^.

### Materials characterization

Microstructural characterizations were performed on a HITACHI SEM operated at 20 kV for the sulfur electrodes with the porosity of 70, 60, and 50%. The representative images are shown in Fig. 1.

### Electrochemical measurement

CR2032 coin cells were assembled in an argon-filled (<5 ppm H_2_O glove box. The as-prepared sulfur electrodes were used as the cathode, lithium metal was used as the anode. The electrolyte was 0.4 M LiTFSI (BASF) and 0.6 M LiNO_3_, 99.95% Sigma-Aldrich) in DME and 1,3-Dioxolane (DOL) (both from BASF, 1:1 by volume). A confined E/S ratio as in 12 μL mg^−1^ is utilized. For sulfur loading of 2.5 mg cm^−2^, 40 μL electrolyte was added. For sulfur loading of 5 mg cm^−2^, 80 μL electrolyte was added. A Maccor Battery Tester was used to perform the galvanostatic cycling measurements between 1.7 and 2.7 V (vs Li^+^/Li^0^) in an environmental chamber at 25 °C. The current density set for tests was referred to the mass of sulfur in the cathode and was set up at 0.05 C for the 1^st^ and 2^nd^ cycle, 0.1 C for the rest of the cycles (sulfur loading of 5 mg cm^−2^). The current density of samples with sulfur loading of 2.5 mg cm^−2^ was 0.1 C from the 1^st^ cycle. Electrochemical impedance spectroscopy (EIS) measurements were performed with a Biologic VMP3 in the frequency range of 1 MHz–0.1 Hz with a sulfur electrode loading of 2.5 mg cm^−2^ at the state of 50% of charge after five cycles.

### Computation details

Classical MD was used to investigate the diffusion coefficient of Li_2_S_4_ in DME solvents. First, amorphous cells were constructed with randomly packed Li_2_S_4_ and DME molecules with different ratios. The exact composition in each solution and the corresponding Li_2_S_4_ concentration in terms of S are listed in Supplementary Table 4. The original density was set to be the same as the solvent (0.90 g cm^−3^). The solution structure was then optimized with the COMPASS2 forcefield^57^. The force field types and the atomic charges are listed in Supplementary Fig. 4 in the supporting information. The optimized cell was subject to a classical MD simulation with NPT ensemble at 298 K and 1 atm for 100 ps to determine the density. The converged densities, calculated as the average density of the last 50 ps, were also listed in Supplementary Table 4. After that, an NVT simulation at 298 K was conducted for 100 ps to track the mean squared displacement (MSD) of both Li_2_S_4_ and DME.

All the classical MD simulations were done through the Forcite modulus in Materials Studio. The timestep was set to be 1 fs. The temperature was controlled by Nosé algorithm^58^ and the pressure was controlled by Berendsen algorithm^59^. An Ewald summation was used for the electrostatic interaction, and the van der Waals interaction was truncated at 15.5 Å.

## Supplementary information

Supplementary Information

Dataset 2, Dataset 3, Dataset s2, Dataset s5

## Data Availability

The authors declare that the data supporting the findings of this study are available within the paper and its Supplementary Information files.

## Supplementary information

**Supplementary information** in available for this paper at 10.1038/s41467-019-12542-6.
