# Supplementary material for: Cathode porosity is a missing key parameter to optimize lithium-sulfur battery energy density
Source: Nat Commun. 2019 Oct 10;10:4597. doi: 10.1038/s41467-019-12542-6 (PMC6787095; doi:10.1038/s41467-019-12542-6)
Supplement: Supplementary file 1 — Supplementary Information [file 41467_2019_12542_MOESM1_ESM.pdf]

Supplementary Information

Cathode Porosity is a Missing Key Parameter to Optimize Lithium-Sulfur Battery Energy Density

Ning Kang[a, ‡], Yuxiao Lin[b, ‡], Li Yang[c], Dongping Lu[d], Jie Xiao[d], Yue Qi[b*], Mei Cai[c*]

a. Optimal CAE, Inc, Plymouth, MI 48170

b. Department of Chemical Engineering and Materials Science, Michigan State University, East Lansing, MI 48824

c. Chemical and Materials Systems Laboratory, General Motors Global R&D Center, Warren, MI, 48092

d. Pacific Northwest National Laboratory, Richland, WA, 99352

‡These authors contributed equally to the work.

*Corresponding author: mei.cai@gm.com; yueqi@egr.msu.edu

Supplementary Methods

Diffusion coefficient calculation: To determine the influence of concentration on Li-PS diffusion, the self-diffusion coefficient D was calculated with classical MD simulation based on the following equation:

$$D = \frac{1}{6N_a} \lim_{\tau\to\infty} \frac{d}{d\tau} \sum_{t=0}^{t-\tau} \sum_{i}^{N_a} [r_i(t+\tau) - r_i(t)]^2 = \frac{1}{6} \lim_{t\to\infty} \frac{d}{d\tau} MSD(\tau) \quad (1)$$

Here t is the simulation time, τ is the time interval. N_a is the total number of atoms in given species. $r_i(t+\tau)$ and $r_i(t)$ is the position of atom i at time $t+\tau$ and time t. The MSD of Li_2S_4 and DME solvent were plotted as a function of time interval τ in Supplementary Figure 5a and 5b. The calculated self-diffusion coefficients D of Li_2S_4 and DME, as well as the composition, Li-PS concentration in terms of S and simulated density of each solution, were summarized in Supplementary Table 3. A typical snapshot 1.9 M and saturated 8.7 M solution were shown in Supplementary Figure 5c and 5d.

The diffusion coefficient of DME in pure DME solvent and 1.9 M solution were 23.1×10^{-10} and 19.1×10^{-10} m^2 s^{-1}, respectively. These values are consistent with the calculated value from Park [1] and experimental value from Hayamizu [2], confirming the validity of the force field and the simulation method. In 1.9 M solution, the self-diffusion coefficient of Li_2S_4 was 5.6×10^{-10} m^2 s^{-1}. With an increase in Li-PS concentration, D(DME) and D(Li_2S_4) both drop dramatically. For the saturated 8.7 M solution and over saturated 15.4 M solution, the self-diffusion coefficient of Li_2S_4 is only 2.7×10^{-10} and 0.6×10^{-10} m^2 s^{-1}, respectively.

Comparison between diffusion distance and pore size: Given the diffusion coefficient, the self-diffusion distance x of Li_2S_4 in the first plateau can be further estimated as

$$x = \sqrt{Dt} \quad (2),$$

where t is the diffusion time. Considering that the first discharging plateau generally contributes 25% of the total theoretical capacity, the diffusion time t can be estimated at 25% of the total discharging time. Given the fact that an C-rate of N means the total discharging time is set to be $1/N$ hours by changing the current density, the estimated self-diffusion distance x was plotted as a function of C-rate in Supplementary Figure 5e. As we can see, either a higher concentration or a

lower C-rate will result in smaller diffusion distance x. For the saturated 8.7 M solution, the estimated distance at a typical 0.1 C rate is 1.55 mm. Even for the oversaturated 15.4 M solution at a C rate as high as 2.5, the diffusion distance is still 0.13 mm. Both values were far larger than the pore size (5~10 μm) in the carbon matrix. That means the Li_2S_4 diffusion in the cathode region is still fast enough in the cathode region, which is against the proposed diffusion mechanism.

Supplementary Figures

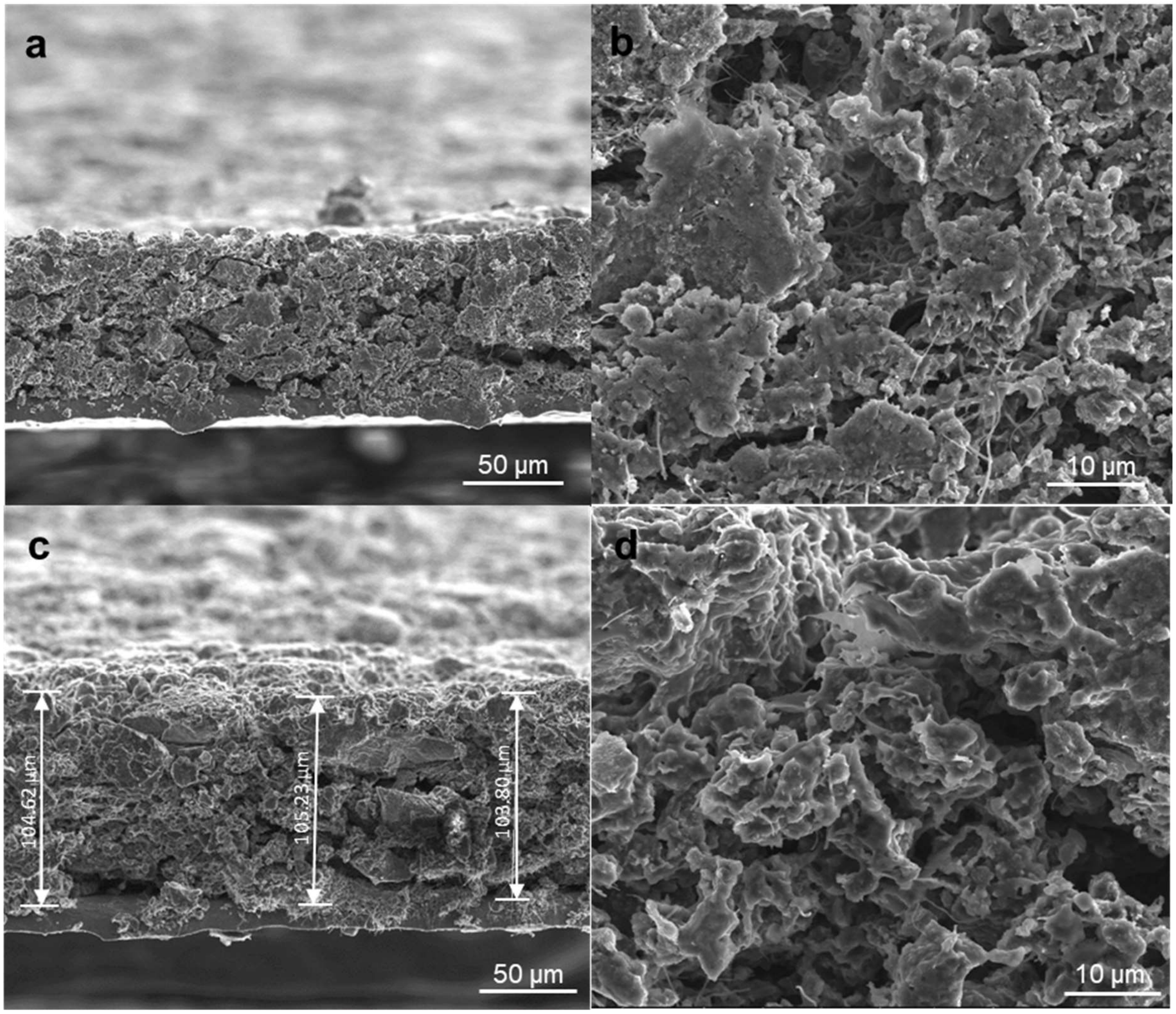

Supplementary Figure 1. Cross section SEM images of sulfur cathode with porosity of 40% (a,b) with initial thickness of 60 μm and 50% (c,d) with initial thickness of 80 μm after cycling

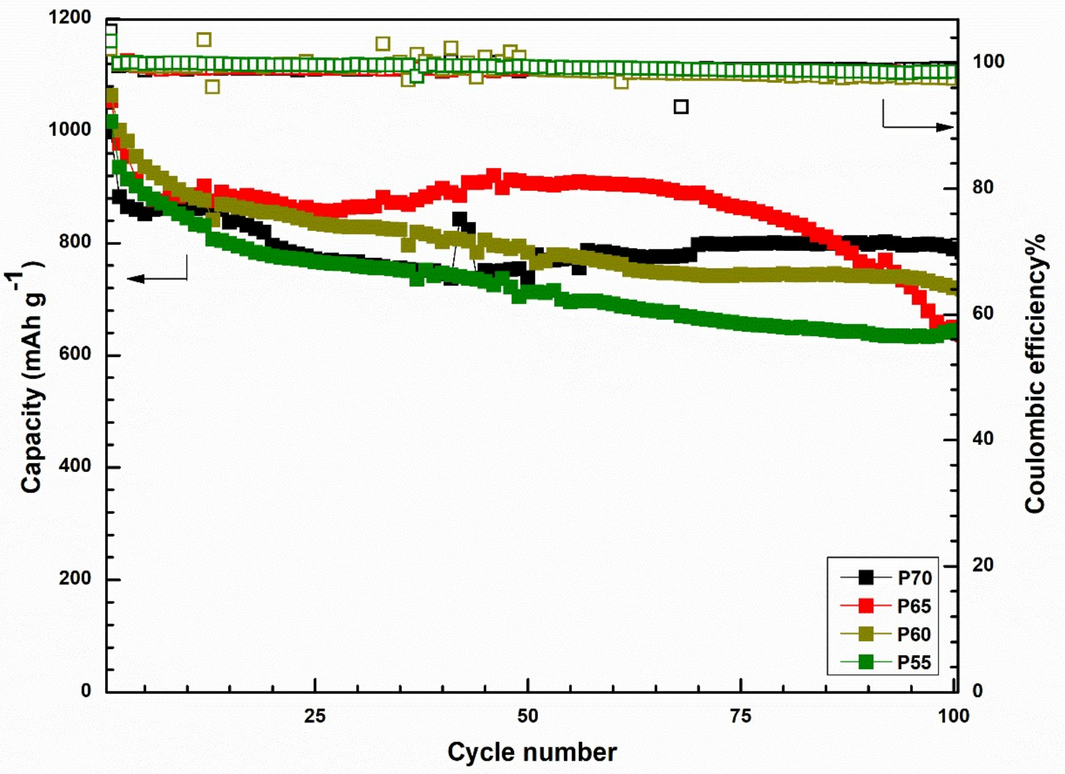

Supplementary Figure 2. Cycle performance of cells with the sulfur loading of 2.5 mg cm^{-2} at a different porosity of 70%, 65%, 60%, and 55%

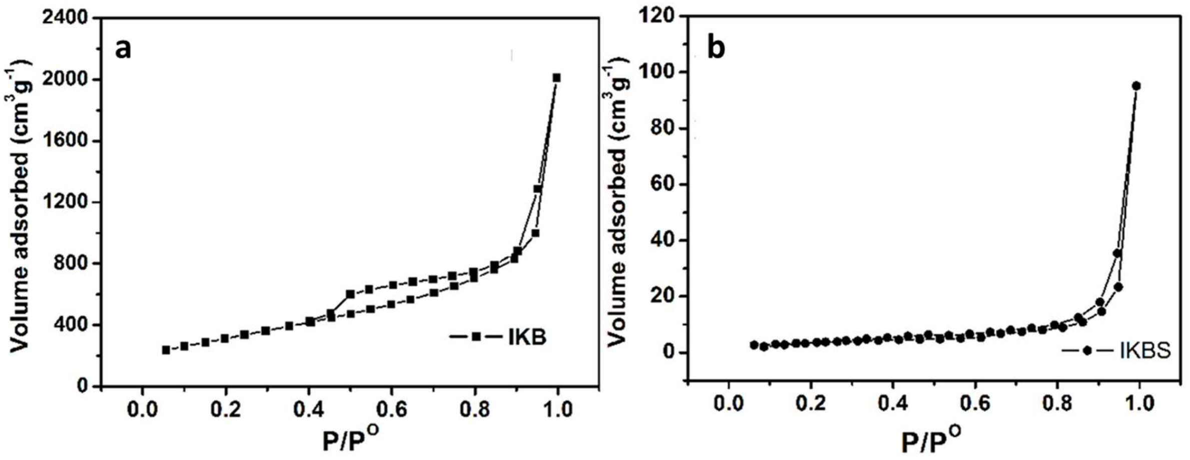

Supplementary Figure 3. Nitrogen sorption isotherms of (a) integrated KB (IKB) with a specific surface area of 1148 $m^2\,g^{-1}$ and pore volume of 3.08 $cm^3\,g^{-1}$, and (b) S/IKB with a specific surface area of 12 $m^2\,g^{-1}$ and pore volume of 0.15 $cm^3\,g^{-1}$.

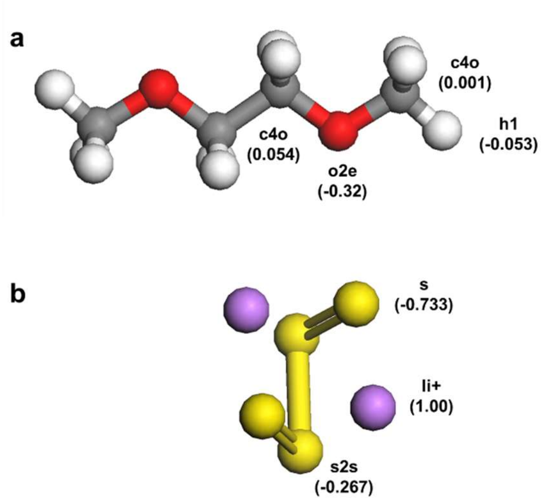

Supplementary Figure 4. The configurations of DME (a) and Li_2S_4 (b) with labeled force field type and charge

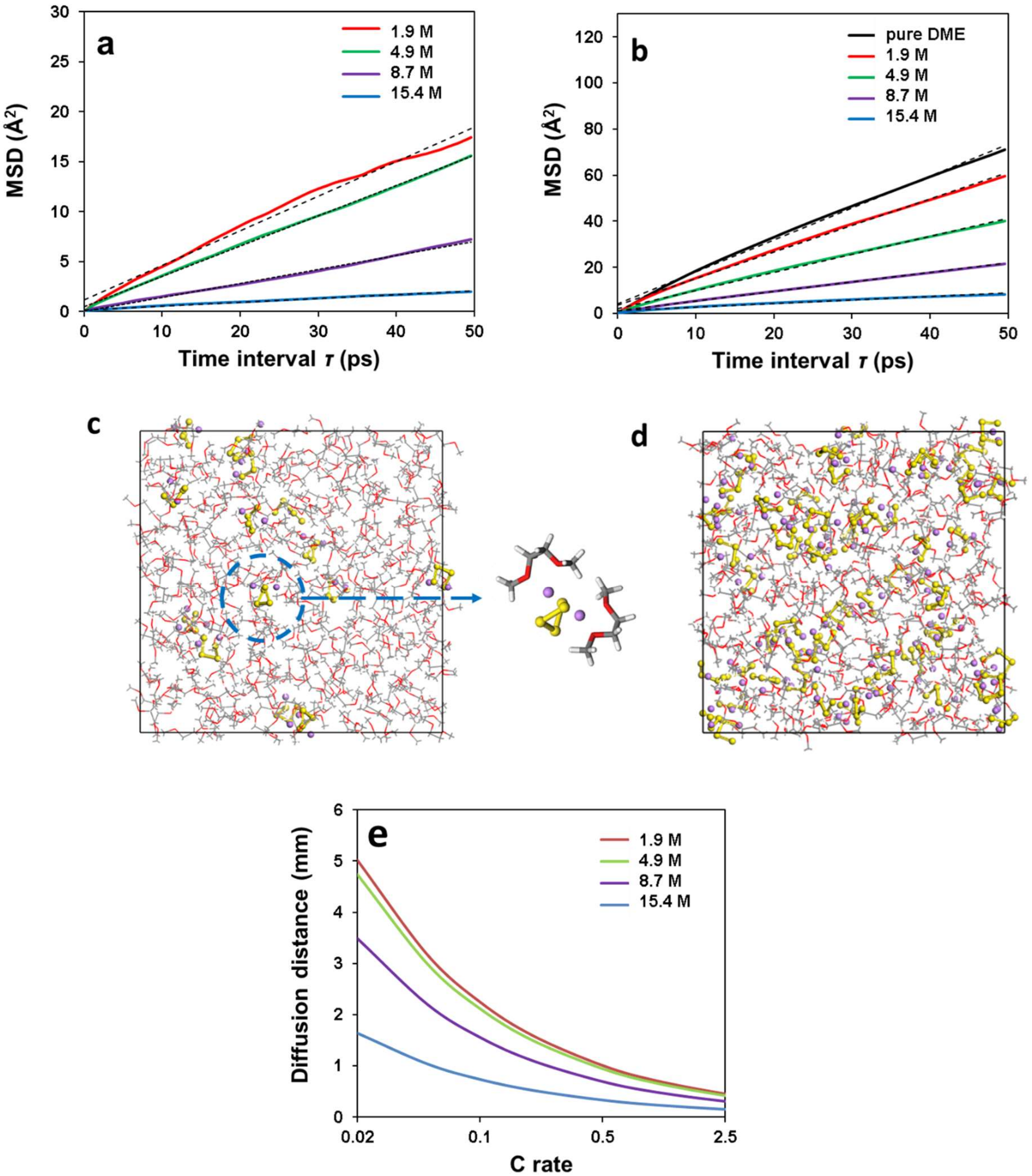

Supplementary Figure 5. Simulated average mean squared displacement (MSD) of Li_2S_4 (a) and DME (b) as a function of simulation time in each solution. A typical snapshot of solution 2.1 M (c) and saturated 8.7 M (d) solution. Estimated self-diffusion distance of Li_2S_4 as a function of C-rate in each solution (e).

Supplementary Tables

Supplementary Table 1. Composition of materials used in the composite cathode

Materials	S	Carbon matrix	Carbon black	CMC-SBR	Total
Mass (mg)	6.50	1.85	1.95	0.65	10.93
Weight percentage (%)	59.1	16.9	18	6.0	100
Compact density (g cm^{-3})	2.07	2.26	1.90	1.90	-
Dense volume (mm^3)	3.14	0.81	1.02	0.34	5.31

Supplementary Table 2. Estimated E/S ratio of coin cell (Unit: µl mg^{-1})

Porosity / Areal capacity (mAh cm^{-2})	70%	60%	50%	40%
1	5.15	4.22	3.68	3.30
2	3.80	2.91	2.39	2.02
3	3.37	2.48	1.94	1.58
4	3.16	2.26	1.72	1.36
5	3.03	2.14	1.59	1.22

Supplementary Table 3. List of parameters

Symbol	Description	Category	Value
V_{pore}	Total pore volume in cathode region	Function of p	-
V_{pore}(sep)	Pore volume in separator	Measured constant	2.5 mm^3
$V_{pore}(cat)$	Pore volume in cathode	Function of p	-
$V_{dense}(cat)$	volume of dense cathode materials without any porosity	Measured constant	5.3 mm^3
p	Porosity	Variable	-
$P_s(uti)$	Utilization percentage of S in the first plateau	Function of p	-
$m_s(uti)$	Mass of utilized S in the first plateau	Function of p	-
$m_s(total)$	Total mass of S in the cathode	Measured constant	6.5 mg
Q_{pr}	Practical capacity in the first plateau	Function of p	-
Q_{th}	Theoretical capacity in the first plateau	Calculated constant	420 mAh g^{-1}
g	Parameter to include extra Li-PS into bulk electrolyte	Matching constant	1.8
S_{max}	Saturated concentration of Li-PS in the electrolyte solvent in terms of S	Measured constant	8 mol L^{-1}
M_s	Molar mass of S	Constant	32 g mol^{-1}
$c_s(uti)$	Practical concentration of Li-PS due to the saturation limit	Function of p	-
A_0	Surface area of carbon matrix per gram	Function of p	-
α	Empirical constant to describe the relationship between A_0 and V_0	Empirical constant	1
A_0	Constant to match the BET measured surface area of carbon matrix with 70 % porosity	Matching constant	300 m^2 g^{-1}
A_{eff}	Effective surface area	Function of p	-
k	Ratio between the mass of non-utilized S and its covering surface area	Fitting parameter	1.27×10^5 m^2 g^{-2}
R	Tunneling resistance caused by the deposited Li_2S_2/Li_2S layer	Function of p	-
C	Parameter to describe the relationship between tunneling resistance and layer thickness. Later combined to C'		-
B	Parameter to describe the relationship between tunneling resistance and layer thickness. Later combined to B'		-
d	Thickness of Li_2S_2/Li_2S layer	Function of p	-
Q	Total capacity in the discharging curve	Variable	-
b	Parameter to describe the relationship between thickens and total capacity. Later combined to B'		-
m_C	Mass of carbon matrix in the cathode	Measured constant	1.85 mg
I	Discharging current, later combined to C'	Measured constant	-
$B'=Bb$	Parameters to describe relationship between discharging voltage and capacity	Fitting parameter	1.07×10^{-3} m^2 g mAh^{-1}
$C'=CI$	Parameters to describe relationship between discharging voltage and capacity	Fitting parameter	0.050 V

Supplementary Table 4. List of composition, density, Li-PS concentration and diffusion coefficient of DME and Li_2S_4 in each solution

Solution	$N(Li_2S_4)$: N(DME)	Density ($g\ cm^{-3}$)	c_S ($mol\ L^{-1}$)	D(DME) ($10^{-10}\ m^2\ s^{-1}$)	$D(Li_2S_4)$ ($10^{-10}\ m^2\ s^{-1}$)
Pure DME	0:300	0.88	0	23.1	-
1.9 M	14:280	0.94	1.9	19.1	5.6
4.9 M	39:299	1.01	4.9	13.0	5.0
8.7 M	60:240	1.10	8.7	6.9	2.7
15.4 M	100:200	1.24	15.4	2.5	0.6

Supplementary Reference

1. Chanbum Park, Matej Kanduč, Richard Chudoba, Arne Ronneburg, Sebastian Risse, Matthias Ballauff, JoachimDzubiella. Molecular simulations of electrolyte structure and dynamics inlithium–sulfur battery solvents. Journal of Power Sources, 2018, 373, 70–78.

2. Kikuko Hayamizu, Yuichi Aihara, Shigemasa Arai, Cirilo Garcia Martinez. Pulse-Gradient Spin-Echo ^{1}H, ^{7}Li, and ^{19}F NMR Diffusion and Ionic Conductivity Measurements of 14 Organic Electrolytes Containing $LiN(SO_2CF_3)_2$. The Journal of Physical Chemistry B, 1999, 103, 519-524.
